# A Simple and Systematic Approach to Qualitative Data Extraction From Social Media for Novice Health Care Researchers: Tutorial

**DOI:** 10.2196/54407

**Published:** 2024-07-09

**Authors:** Kelly Pretorius

**Affiliations:** 1 School of Health Sciences St. Edward's University Austin, TX United States; 2 School of Nursing Indiana University Indianapolis, IN United States; 3 Pediatric Hospital Medicine Texas Children's Hospital North Austin Austin, TX United States

**Keywords:** social media analysis, data extraction, health care research, extraction tutorial, Facebook extraction, Facebook analysis, safe sleep, sudden unexpected infant death, social media, analysis, systematic approach, qualitative data, data extraction, Facebook, health-related, maternal perspective, maternal perspectives, sudden infant death syndrome, mother, mothers, women, United States, SIDS, SUID, post, posts

## Abstract

Social media analyses have become increasingly popular among health care researchers. Social media continues to grow its user base and, when analyzed, offers unique insight into health problems. The process of obtaining data for social media analyses varies greatly and involves ethical considerations. Data extraction is often facilitated by software tools, some of which are open source, while others are costly and therefore not accessible to all researchers. The use of software for data extraction is accompanied by additional challenges related to the uniqueness of social media data. Thus, this paper serves as a tutorial for a simple method of extracting social media data that is accessible to novice health care researchers and public health professionals who are interested in pursuing social media research. The discussed methods were used to extract data from Facebook for a study of maternal perspectives on sudden unexpected infant death.

## Introduction

The Pew Research Center began tracking social media use in 2005, at which time only 5% of the American population reported its use [1]. As of 2021, social media use was considered widespread, with approximately 7 in 10 Americans reporting its use [2]. Social media is defined by Merriam-Webster as “forms of electronic communication (such as websites for social networking and microblogging) through which users create online communities to share information, ideas, personal messages, and other content (such as videos)” [3]. The following platforms are considered social media per the Pew Research Center: Facebook, Pinterest, Instagram, LinkedIn, Twitter (subsequently rebranded as X), Snapchat, YouTube, WhatsApp, Reddit, TikTok, and Nextdoor [2]. With the growth of social media use, users’ demographics have also become more representative of the US population [1]. Social media is also commonly accessed by patients to discuss and obtain information about health [4], especially among the younger generation [5].

The widespread use of social media has therefore created vast amounts of data for health care researchers to leverage to understand health problems. Such data are beneficial for not only health care researchers but also public health practitioners and those employed in the public health sector. For instance, health departments or local governments might find it valuable to analyze social media data to identify local health care needs or perspectives within specific populations they serve. This is because social media analyses offer several advantages, such as providing insight into the lived experiences of people with specific health conditions [6]. Additionally, because social media allows for open and honest communication among its users, analyzing social media content has led to the identification of new themes and concerns among populations that had previously gone undiscovered [7,8].

The methods for acquiring social media data for health care research are continually evolving, with ongoing advancements in machine learning techniques for collecting and analyzing such data [9]. Thus, some techniques used for social media analyses include but are not limited to natural language processing, news analytics, opinion mining, scraping, sentiment analysis, and text analytics [10]. For example, studies have obtained data from Twitter via text mining [11,12], and natural language processing has been used to identify posts of interest on Facebook [13]. Other studies have obtained data by searching and extracting information from Facebook [14] or obtained data on health conditions by searching Facebook pages using a generic Facebook account [15]. Recent social media analyses on Instagram describe identifying posts using specific hashtags [16]. Another Instagram study mentions the use of an open-source web scraper to identify photos for analysis [17]. Lastly, studies have used Word Adjacency Graph modeling, a social network analysis, to better understand chronic disease processes [18,19]. The most popular method of obtaining social media data is via text mining [20], where researchers use software to scrape data from social media platforms [10].

While some social media data extraction and analysis software tools are open source, specialized software is often required, which can be costly [10]. Furthermore, specialized software is not readily available for all health care researchers and is sometimes incompatible with certain platforms. For example, there have been times when social media platforms restricted data scraping [21], and accessing raw data from Google and Facebook is becoming increasingly difficult [10]. Additionally, despite their popularity, challenges related to the techniques discussed above have been identified [22] and include issues related to handling slang, spelling errors, foreign words, and the general evolution of language [10]. Language obtained via social media can provide incredible insight into individuals, including behavioral processes; yet language changes over time and can be noisy and ambiguous, and capturing “the oddities of social media expression” is therefore challenging [22].

To leverage the potential of social media data and overcome some barriers related to social media research, this paper describes a simple and systematic approach to extracting qualitative data from a social media platform. The methods outlined in this paper were used to extract data from Facebook for a study on maternal perspectives on sudden unexpected infant death (SUID). The purpose of this paper is to provide guidance on extracting social media data without specialized software for novice health care researchers and persons in the public health sector. This is essential so that so all persons, regardless of funding, are provided with the steps to conduct basic social media research.

Before beginning the processes outlined in this paper, researchers should consult their institutional review boards (IRBs) to determine whether their planned study and resulting analysis require board review and approval. Additionally, ethical implications regarding social media research should be considered and are briefly discussed below.

## Ethical Considerations

Given the complexity and breadth of ethical implications in social media research, a comprehensive discussion of the ethics falls beyond the scope of this paper. However, it is critical to acknowledge their significance and discuss their implications briefly. Recent controversies in social media research have resulted in calls for the scientific community to take charge in ensuring ethical use of social media data [23]. For example, the creation of technology ethics boards has been recommended to help guide researchers [23]. Other initiatives have also taken place, such as the creation of the Connected and Open Research Ethics initiative, which brings together stakeholders to guide digital health research [24]. Health care researchers interested in social media research are encouraged to educate themselves on navigating ethics in social media research. Furthermore, the IRB can provide some guidance and assistance regarding ethical considerations. The IRB process will differ significantly based on the proposed research. For instance, the IRB consent processes will differ based on the social media platform; if the proposed data set originates from a medium or format that is preexisting, open or closed; and if the data are publicly available. Additionally, using data from specific social media platforms may become restricted for research, and legal ramifications may result. Despite these considerations, no matter the methodological approach or IRB status (exempt or nonexempt), the protection of the participants’ identity should be at the forefront and is of utmost importance.

The study discussed in this paper was submitted for review at the University of Texas IRB and deemed exempt as the study did not meet criteria for human subjects research. This exemption was given because the study used publicly available data sets. Despite this exemption, rigorous measures were undertaken to safeguard the confidentiality of all participants. Personal and identifying information was meticulously removed from the data set to maintain participant anonymity. Additionally, in discussing the study’s findings, only essential details were shared to ensure participant protection and prevent identification of participants. In describing the steps of data extraction for this paper, care was taken to continue to protect participants’ privacy. Therefore, images of the original data will not be shared in this paper and the included figures have been heavily redacted.

As with any health care research, and despite planned efforts to safeguard participants, there were unforeseen ethical implications in the study discussed. In analyzing the identified data set, new and surprising findings were identified, which is often the case in social media analyses. For example, there were numerous comments shared among users that potentially indicated symptoms of postpartum anxiety or depression. Many of these comments were months old, and some of the members no longer active in the group; thus, the research team was not able to assist participants who were potentially displaying symptoms of postpartum anxiety or depression. Ultimately, the necessity of a preexisting, comprehensive guideline on managing sensitive information became evident during the analysis process. Such a document could have provided direction to researchers when encountering potentially sensitive situations during the analysis. It is therefore recommended that persons conducting social media research, no matter the platform, IRB status, or topic being researched, have a plan for handling sensitive information and specific resources to provide to users, if warranted.

## Steps for Social Media Data Extraction

Textbox 1 provides a concise overview of the steps involved in the selection and extraction of social media data using the outlined method. The specific steps used in the study discussed will be elaborated on below.

**Overview of steps for social media data selection and extraction.**

**Step 1: Select a social media platform**

- Identify a social media platform commonly used by the identified population of interest
- Consider data on user demographics for different platforms (ie, Pew Research Center) [25]

**Step 2: Identify data of interest within the social media platform**

**Figure 1:**
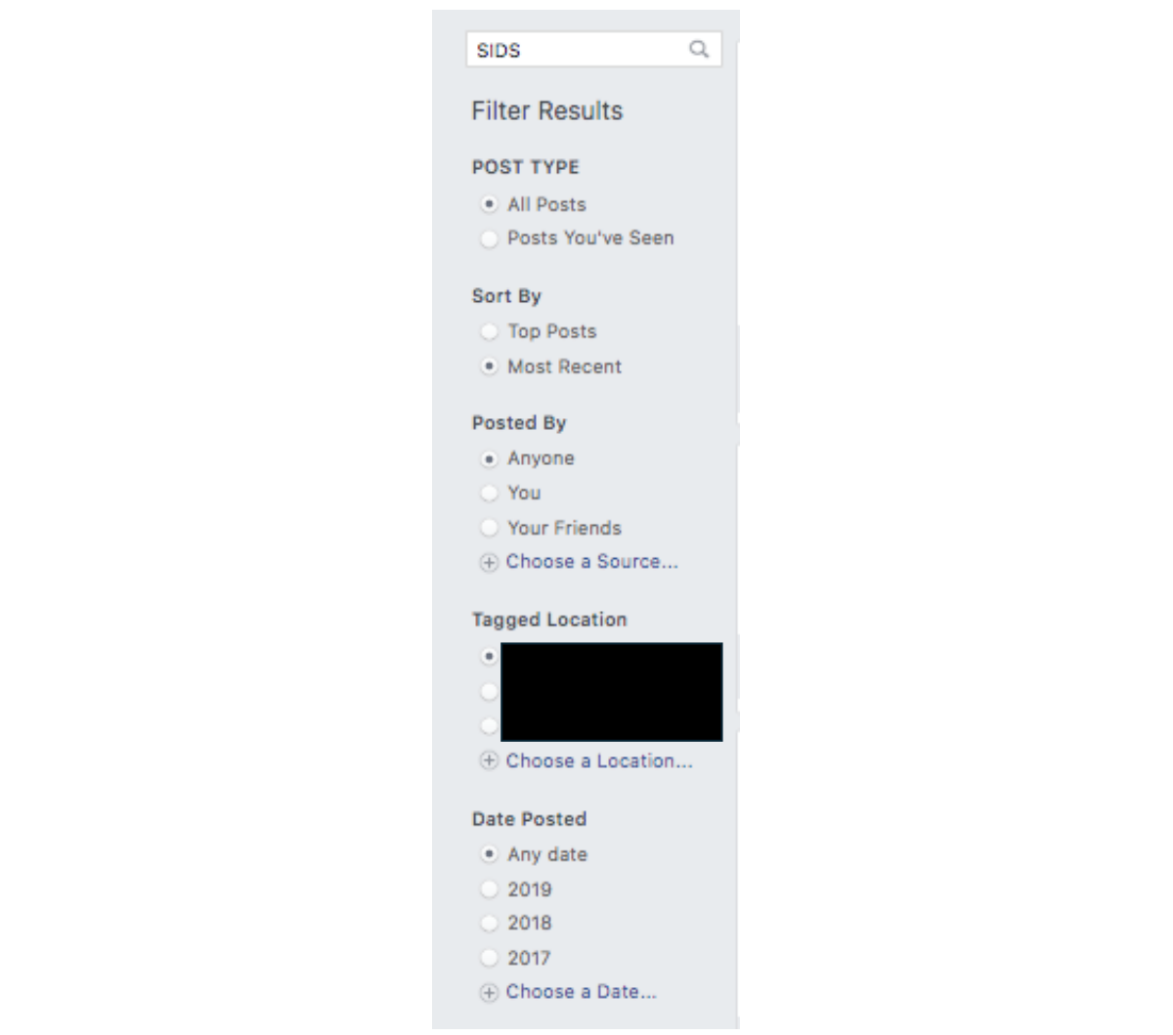
Search toolbar used in the study. SIDS: sudden unexpected infant death.

- Identify data to extract by trialing different searches (Figure 1):
  Consider searching for social media groups formed based on the topic of interest or population of interest (ie, health conditions and support groups)
  If applicable and ethical (and potentially approved by the specific group), consider searching within a specific group on the selected social media platform
- Once the data have been identified on the specific platform (and potentially within a specific group), determine search terms that identify the most relevant conversations or discussions that will address the research aims or questions
- If the resulting data set is too vast, consider applying filters as needed (ie, limit results to the most recent year, or a specified location, if possible or applicable)

**Step 3: Capture data at a specific time point**

- Regardless of institutional review board status, a detailed data management plan should be in place regarding appropriate data storage and destruction of study materials
- After the relevant social media data have been identified and filtered accordingly, capture images and screengrabs to ensure accuracy
- Social media data change frequently and quickly, so this process should be performed in a timely manner
- In this step, it is essential that any identifying information be redacted to protect participant confidentiality
- This data set should be stored on secure and private software to maintain confidentiality and protect participants

**Step 4: Transfer and organize identified data**

**Figure 2:**
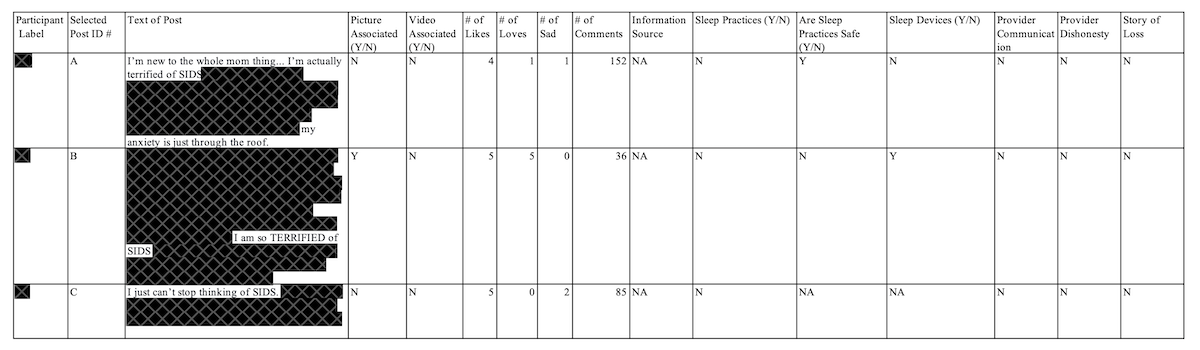
Partial display of the data extraction tool and main posts, heavily redacted to maintain participant confidentiality. N: no; NA: not applicable; SIDS: sudden infant death syndrome; Y: yes.

**Figure 3:**
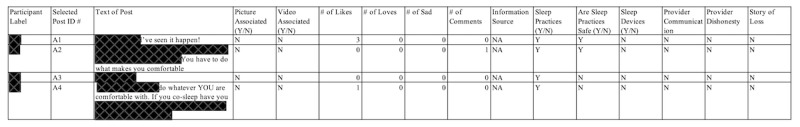
Partial display of the data extraction tool and subcomments, heavily redacted to maintain participant confidentiality. N: no; NA: not applicable; Y: yes.

- After the data have been time-stamped, the identified data should be transferred to data management software (ie, REDCap [Research Electronic Data Capture]) to facilitate further analysis
- A data extraction tool (Figures 2 and 3) should be created before the data extraction process
- The data extraction tool should include information needed to address the research aims or questions
- In this step, it is essential that any identifying information be redacted and not transferred to the data management software to protect participant confidentiality
- This data set should be stored on secure and private software to maintain confidentiality and protect participants

**Step 5: Ensure accuracy of data set**

- After transferring data to data management software, compare the time-stamped images of the data with the data extracted
- In this step, it is essential to continue to check for and remove any identifying information to protect participant confidentiality
- Maintain a thorough audit trail by journaling and keeping a detailed log regarding this process

**Step 6: Format data set for analysis**

- Consider using qualitative data analysis software to complete the analysis
- Once the analysis software is selected, adjust the format as needed to facilitate this analysis
- Finally, complete the analysis of the identified data set per the selected methodology

### Step 1: Select a Social Media Platform

Selecting a social media platform is the first step in conducting a social media analysis. Different social media platforms are more common among various demographics; thus, the selection of the social media platform should be considered and justified by the research team. The Pew Research Center provides detailed statistics on demographic use and popularity of many social media forums [26]. For example, 52% of Hispanic and 49% of Black Americans use Instagram, versus only 35% of White Americans [2]. Women are more likely to use Pinterest compared with men, and persons with a higher level of education tend to use LinkedIn [2]. Differences also exist among age groups: 84% of adults between the ages of 18 and 29 years use social media, while only 45% of those aged 65 years and older use social media [2]. One platform may therefore allow better insight into a specific population of interest when compared with another.

For the study discussed, the population of interest was birthing persons. At the time of the study and according to the Pew Research Center, Facebook is one of the most popular social media formats among those of childbearing age [26,27]. Furthermore, Facebook is popular among mothers who are seeking parenting information [28] and is unique in terms of its various groups, such as Facebook mother’s groups, in which the members can participate. Given all these points, Facebook was selected as the social media platform for the study.

### Step 2: Identify Data Within the Social Media Platform

After selecting the social media platform that aligns with the target population, the next step is to identify the data to be extracted for subsequent analysis. In the discussed study, the goal was to identify a specific group on the selected platform that would yield sufficient conversations surrounding the health topic of interest, SUID. After searching many Facebook groups, the group that was selected for data extraction was for women, based in the United States, and had many members (approximately 17,500 at the time of selection). Although not all birthing persons identify as women or mothers, the data set originated from individuals who self-identified as “mothers.”

Once in the selected group, to identify qualitative data for extraction and analysis, existing conversations among group members were searched using the search toolbar feature (see Figure 1). After trialing various search terms, the term that resulted in the most “posts” and relevant conversations about the topic of interest was “SIDS” (sudden infant death syndrome). Other terms, such as “SUID” and “ASSB” (accidental strangulation or suffocation), related to this concept are popular among health care researchers and providers; however, parents frequently use the term “SIDS” when discussing safe sleep and SUID. While Facebook allows additional search filters to be applied, none were applied during this step. The purpose of this was to yield the most data for extraction. Specifically, the following options were selected: “all posts,” “most recent,” posted by “anyone,” tagged location of “anywhere” and “any date” posted. In the study discussed, applying no filters and searching for “SIDS” in the toolbar resulted in 20 unique posts, with 912 additional comments that were relevant to the topic of interest and addressed the study’s research questions. A total of 512 individual participants engaged in the selected data set.

If another social media platform were chosen or if a larger Facebook group were selected, searching conversations for a specific topic may have resulted in excessive or overwhelming amounts of data. In this situation, the research team may choose to alter or apply filters, such as limiting the results to more recent years. If the data set is still too large, the research team may choose to extract and analyze a random sampling of the available data. In selecting the data to be extracted and analyzed, researchers need to trial sampling methods to ensure that research questions can be addressed by the selected data. Only once the sampling plan has been established should the research team proceed with data extraction.

### Step 3: Capture Data at a Specific Time Point

After identifying the social media data to extract and analyze, the next step is to time-stamp the data. Social media data evolve daily, particularly on platforms such as Facebook. For example, members may edit a post, add a comment, or respond to other posts at any time. Steps were therefore taken to maintain a detailed and time-stamped record of the data to be analyzed.

After identifying the data, images of the posts and related conversations were taken. It is important that the researcher carefully read the posts and related conversations to ensure that all comments are fully expanded and to redact any identifying participant information. Facebook, for example, automatically shortens longer comments and posts. To protect the identity of the participants, names and any identifying information were redacted on the images, and labels were assigned for each participant. Images of the 20 posts were then organized and labeled. The images were categorized in alphabetical order: post 1 was labeled “A,” and subsequent posts were sequentially labeled “B,” “C,” and so forth. Subsequent conversations below the posts were labeled “A1,” “A2,” and so forth to allow the researcher to understand comments and subconversations related to the main posts.

Images of the posts and comments with redacted identifying information were then saved to a secure university computer and uploaded to a secure university website. For this study, this process took approximately 12 hours and was completed in 1 day to capture the data at that specific time.

### Step 4: Transfer and Organize the Data

After capturing social media data at a specific time point, the next step is data extraction and organization. In this study, the selected data were transferred from the Facebook page and organized within data management software. REDCap (Research Electronic Data Capture; Vanderbilt University) is recommended as a data management software option for this process to ensure a transparent data management workflow. Again, to protect the identity of the participants, names and any identifying information were redacted on the transferred data. Spreadsheets were organized by post (in total there were 20), and the posts were again labeled in alphabetical order (A to S; see Figure 2). Each post’s comments were then labeled “A1,” “A2,” and so forth (see Figure 3). To enable the researchers to follow the conversations, the deidentified labels assigned to participants were also transferred. Because many comments on Facebook resulted in subconversations, each subconversation was labeled “A1a,” “A1b,” and so forth, to allow the researcher to understand when a participant may be responding to a comment versus the original post. Maintaining a detailed record of these particulars was essential for the analysis and in understanding the true meaning of the data. This process took approximately 2 weeks to complete.

The data extraction spreadsheet also contained columns for information related to the study’s research questions (see Figure 2). For example, the study collected additional information on if there was a picture associated with the comment, if there was a video associated with the comment, and various other questions related to SUID and infant sleep. This information was collected on all posts, comments, and subcomments.

### Step 5: Ensure Accuracy of Data

To ensure accuracy of the data, after the data were copied to the selected data management software, the data set was manually compared with the images. To maintain an audit trail, any corrections to the transfer errors that were identified during this comparison were recorded. We recommend maintaining detailed memoing and documentation throughout the entire data extraction process to ensure a complete audit trail. Memoing involves chronical documentation of the research journey and allows for deep engagement with qualitative data; writing detailed memos throughout the qualitative process is an effective way to enhance qualitative methodologies [29]. The process of checking the accuracy of the data extraction also allows the researcher to ensure that all personal or identifying information is removed to maintain confidentiality of participants. For the study discussed, the process of checking the data for accuracy and maintaining an audit trail took approximately 2 days to complete.

### Step 6: Format Data for Analysis

After ensuring accurate data extraction, the next step involves formatting the extracted data for subsequent analysis via various software programs. In this study, the data set was converted to 20 documents based on the 20 main posts to enable qualitative descriptive content analysis via data analysis software, ATLAS.ti [30]. Again, it is important to maintain the participants’ labels and text label from the data management spreadsheet during any format change to allow the researchers to follow the full conversations and subconversations.

The resulting analysis was completed by having 2 coders individually code the entire data set. To assist in the coding, a codebook was created after 25% of the data were coded by the primary researcher; this took place once the coding became redundant. First cycle coding [31] was completed via dialogical intersubjectivity, and 2 researchers also completed the extraction spreadsheet tool, with discrepancies being resolved with discussion. The codebook was adjusted, as needed, and themes were derived after second cycle coding [31]. The resulting analysis and findings have been published [32].

## Discussion

### Strengths of This Approach

There are many strengths of this approach of data extraction from social media platforms. This method can be completed by any health care researcher, whether they have access to specialized software or not. Thus, it could be applied by researchers working in a variety of settings, in many geographic locations, and with various levels of funding. This process may also prove useful among public health practitioners and those in the public health sector. For example, health departments or local governments may need to identify health care needs or viewpoints among specific populations they serve. Researchers may also find the described approach useful in triangulating findings from other research projects, or to better understand a specific population’s discussion about health topics.

Although the described data extraction method is time intensive, it allows for an all-encompassing analysis of the selected material. While data mining methods are popular [20], using a data mining approach may potentially miss essential elements due to the challenges associated with this extraction technique [10]. Many conversations that occur on social media have specific language, emojis, videos, or misspellings [20] that are easier to understand within the entire context. For instance, this Facebook study identified specific terminology and phrases that were unique to the population of interest. Some of these terms and phrases could have been missed or misunderstood if not analyzed in the context of the entire data set or if other extraction techniques were used. Hence, a significant strength of this approach lies in analyzing conversations within their original context, enabling researchers to immerse themselves fully in the data. Interpreting conversations in this way also enables the research team to fully understand the original intent of the user.

Furthermore, this approach of social media extraction allows health care researchers to obtain data without imposing the researcher’s presence, thus increasing the likelihood of acquiring “honest” data. This, again, has been demonstrated in prior research [7,8] and was also supported by the findings from the discussed study [32].

### Limitations in This Approach

While there are strengths to the method described, it is not without limitations. This method, along with other social media data extraction methods that use software, may not be ethical in certain circumstances. Again, health care researchers must educate themselves on the complexity of ethics regarding social media research and should consult with their local IRB before beginning any social media research.

Additionally, health care researchers, whether in academia or in the public health sector, need to understand their population of interest and what social media platform their population gravitates toward. If a population does not use social media, then this method will not yield worthwhile results. For instance, if the population of interest is geriatric, this approach may not be as helpful as in a teenage or younger adult population [2,5].

This approach is also time-consuming compared with other methods of social media data extraction. For instance, data mining may yield quicker results. The amount of data to extract is another limiting factor to this approach. Because the discussed method is labor-intensive, it is not possible to perform this approach when dealing with copious amounts of raw social media data. This approach is more feasible when the researcher has specific research questions for a specific population or is interested in a specific health issue.

Additionally, obtaining data via this method limits the generalizability of the findings because demographic information from users is not obtained. Lastly, this method has not been sufficiently evaluated in comparison with other social media extraction methods. Future social media research should consider a comparison of findings of data obtained via machine learning versus this manual method.

### Conclusions

In conclusion, by breaking down the extraction process into 6 steps, this paper aims to provide a blueprint for novice health care professionals and those in the public health sector to obtain social media data for basic social media research. Although the described process was used to extract data from a Facebook group, this method can be applied to other social media platforms. Despite some of the limitations and ethical considerations, this approach to extracting social media data is worthwhile given the potential discovery of novel information from specific populations about specific health concerns or issues.

It is therefore beneficial for health care researchers and persons serving in the public health sector to recognize the potential use of social media analyses in their respective areas of research and work. Because social media is incredibly popular among the general American population [1] and most demographics [2], the possibilities of conducting social media research are vast, as are the applications of this extraction method.

## Abbreviations

IRB: institutional review board
REDCap: Research Electronic Data Capture
SIDS: sudden unexpected infant death
SUID: sudden unexpected infant death
